# Influence of Place of Birth on Adult Mortality: The Case of Spain

**DOI:** 10.1007/s10680-023-09679-y

**Published:** 2023-09-07

**Authors:** Néstor Aldea, Dariya Ordanovich, Alberto Palloni, Diego Ramiro, Francisco Viciana

**Affiliations:** 1 Institute of Economy, Geography and Demography (IEGD), CSIC-CCHS, Calle Albasanz 26-28, 28037 Madrid, Spain; 2 French Institute for Demographic Studies (INED), Aubervilliers, France; 3 Institute of Statistics and Cartography of Andalusia (IECA), Seville, Spain; 4 Center for Demography & Ecology, University of Wisconsin-Madison, Madison, WI, USA

**Keywords:** Place of birth, Geographic inequalities, Mortality, Internal migration

## Abstract

We use a unique data set from Spain and we estimate life expectancy at age 50 for males and females by place of residence and place of birth. We show that, consistent with expectations regarding the influence of early conditions on adult health and mortality, the effects of place of birth on adult mortality are very strong, irrespective of place of residence. Furthermore, we find that mortality levels observed in a place are strongly influenced by the composition of migrants by place of birth. This is reflected in a new measure of heritability of early childhood conditions that attains a value in the range 0.42–0.43, implying that as much as 43 percent of the variance in Spain’s life expectancy at age 50 is explained by place of birth. Finally, we find evidence of the healthy migrant effect, that is, positive health selection of migrants, at a regional level.

**Supplementary Information:**

The online version contains supplementary material available at 10.1007/s10680-023-09679-y.

## Introduction

Significant geographic disparities in adult mortality by place of residence are ubiquitous and well entrenched in high-income populations (Boyle, 2004; Wilmoth et al., 2011). These disparities may reflect the impact of a combination of factors characteristic of places of residence, including income (Chetty et al., 2016), exposures to hazards (air and water pollution (Dwyer-Lindgren et al., 2016; Murray et al., 2006; Rogerson & Han, 2002), public infrastructure (Ezzati et al., 2008), health and medical care service (Finkelstein et al., 2021), income inequality (Wilkinson & Pickett, 2008), and socioeconomic mobility (Venkataramani et al., 2020). In most populations studied so far, regional disparities are a persistent feature as is the ranking of mortality levels that places exhibit. Data from the USA, for example, indicate that the magnitude of disparities across states is as large as 8 years of life expectancy at birth (Ezzati et al., 2008; Murray et al., 2006; Wilmoth et al., 2011). Much larger gaps are found at lower levels of aggregation (counties) as they span values between 0 and 20 years (Kindig & Cheng, 2013; Kulkarni et al., 2011).

In Spain, the country we study in this paper, as well as in a handful of other high-income countries studied in previous research (Boyle, 2004; Wilmoth et al., 2011), regional mortality disparities are less sharp than in the USA but still quite large. As of 2003, the gap in life expectancy at birth across Spain’s provinces was of the order 5.1 years, whereas in Autonomous Communities (the largest aggregate units) the gap was about 2.6 years (INE, 2022).^1^ As in the USA, these disparities are attributed to the influence of contextual characteristics of places of residence. Indeed, with a few exceptions (Fletcher et al., 2023; Xu et al., 2020), the determinants of disparities identified by standard research, are always contextual or aggregated individual attributes, such as income and education, of places of residence. The impact of composition of the population by migrants’ origin and their health characteristics are given short shrift. This practice is generalized since most deaths and population data only identify individuals’ place of residence at the time the information is collected. As a consequence, the only accounting possible is one in which observed disparities are imputed to differences in contextual (or aggregated individual) traits of places of residence. This is a risky default explanation as some disparities may be the outcome of regional heterogeneity in the composition of individuals by life history exposures, all of which are concealed from view in this type of data.

To overcome some of these shortcomings, we use a unique data set from Spain which avails us with information on mortality by both place of birth and place of residence. As we argue below, and as shown by recent research, complementing information on mortality by place of residence with information on mortality by place of birth, can significantly enrich the accounting of regional mortality disparities (Fletcher et al., 2023; Xu et al., 2020).

The paper has two goals. First, we estimate life table functions by place of birth and place of residence and illustrate the extent to which these differ from each other. The second goal is to address the problem posed by health selection and, simultaneously, verify hypotheses from the Developmental Origins of Adult Disease (DOHaD) paradigm.^2^ We propose a model that decomposes life expectancy into factors associated with place of birth, residence, and selection effects. We also propose a simple expression to estimate the magnitude of ‘heritability’ of place of origin conditions, e.g., the degree to which migrants’ mortality in places of residence resembles mortality experienced in places of birth. This heritability measure is an indicator of the extent to which exposures during early life in places of birth partially manifest, as the DOHaD paradigm would predict, in older adult ages mortality in places of destination.

## Background

Under conditions of very low or no residential mobility, some of the many candidate contextual traits in places of residence may be sufficient to account for the whole of mortality disparities across places. But doing so in populations with significant residential mobility could be misleading.^3^ Thus, for example, in the USA about 30% of 2010 adults resided in a state they were not born in. Spain is also a country with important flows of internal migrants as 28% of the population aged between 50 and 82 in 2003 reside in Autonomous Communities (largest aggregation units) they were not born in. Similar conditions characterize Canada, Germany and France (Wilmoth et al., 2011). In all these cases, observed mortality disparities may include a non-negligible component associated with conditions experienced by migrants early in life that might be reflected in individuals’ place of birth.

### New Accounting of Regional Mortality Disparities

Ignoring the foregoing shortcomings of standard mortality statistics can be consequential for two reasons. First, in most cases, the population distribution by place of residence is shaped by past migration flows. Second, there is abundant empirical research demonstrating that individuals’ adult health and mortality risks are influenced by their life course exposures and, in particular, their experiences during early childhood (Ben-Shlomo et al., 2014; Gluckman, 2006; Gluckman & Hanson, 2004; Haas, 2008; Hayward & Gorman, 2004; Kuh & Shlomo, 2004). An improved analytic strategy is to complement information on mortality by place of residence with information on mortality by place of birth. The latter is an indicator, albeit imperfect, of experiences during an important stage of individuals’ life history, namely, soon after birth and during early childhood.

### Healthy Migrants, Salmon Bias and the Enduring Legacy of Early Exposures

Two factors account for the interplay between residential mobility and observed regional mortality. The first is reverse causality, e.g., residential mobility is partially dependent on health status. Its relevance is a function of how health selection shapes the composition of immigrants in regions of destination. This is known as “healthy migrant effect”, one whereby migrant populations tend to have, at least soon after migration, more favorable health and mortality profiles than large swaths of the population at destination (Abraído-Lanza et al., 1999; Palloni & Arias, 2004; Palloni & Morenoff, 2001; Turra & Elo, 2008). One explanation for this pattern is that, except for some refugee populations, immigrants are a draw from subgroups at origin with better health status at the time of migration. Conversely, the so-called salmon bias (Abraído-Lanza et al., 1999; Dunlavy et al., 2022; Palloni & Arias, 2004; Palloni & Morenoff, 2001; Turra & Elo, 2008) refers to a phenomenon whereby immigrants return to the place of origin as a consequence of ill-health. The existence of a healthy migrant effect will produce two results: It will improve the average (and perhaps increase the variance) of health status and mortality risks at destination and, almost surely, will diminish those at origin. Different places harbor migrant populations from different origins, of different size, and are, therefore, characterized by variable health selection. We should then expect that at least a fraction of observed regional disparities by place of residence, e.g., those embedded in traditional measures of mortality, could be rooted in the directionality and size of migration flows. On the other hand, the impact of the “salmon effect” induces reverse health selection and should also have an impact on observed regional disparities. Its magnitude will depend on the prevalence of the selection mechanism and the places most compromised by it.

The second factor associated with residential mobility that could account for regional mortality disparities is the strength of the association between early conditions, adult health and mortality, and migration flows (Galobardes et al., 2004, 2006; Hamad et al., 2016; Hayward & Gorman, 2004; Palloni et al., 2009). This association could either reinforce or dilute observed mortality disparities by place of residence. Most migrants are accompanied by kin and carry with them material possessions. They also carry personal profiles, including their genetic make-up, habits, preferences, and learned behaviors (Deryugina & Molitor, 2020). Importantly, they are carriers of latent risks associated with exposures during early life whose effects are largely manifested post-migration, after attaining some critical adult ages.

In the last 20 years or so, a growing body of research under the so-called Developmental Origins of Health and Disease (DOHaD), has produced empirical evidence supporting a conjecture that was originally posited by Barker (1990, 1998) and later reformulated and extended by Gluckman, Hanson and colleagues (Bateson & Gluckman, 2012; Bateson et al., 2014; Gluckman, 2006; Gluckman & Hanson, 2004; Langley-Evans, 2004). According to it, individuals may be exposed in utero or during infancy and early childhood, to adverse conditions, including nutritional constraints, stressful contexts, or toxic environments. Despite these adverse conditions, they may survive to attain adult ages as a result of plastic adaptations of the embryonic/fetal growth and development plans. However, survival to adulthood may come at a steep price, in the form of inheritance of enhanced risks of chronic illnesses, including obesity, metabolic syndrome, Type 2 Diabetes (T2D), hypertension, coronary artery disease (CAD), stroke, and cognitive decline. These are precisely the conditions that account for the bulk of mortality in modern human populations and, therefore, should also explain the bulk of regional mortality differentials. The onset of these chronic conditions usually occurs after attaining critical ages and we will refer to their impacts as “adult delayed effects”. If places’ mortality levels and prevalence of adverse conditions are correlated and, in addition, the DOHaD’ conjecture is correct, individuals born in places with higher mortality will also experience comparatively longer bouts and higher levels of adverse early conditions that manifest as adult delayed effects.^4^

The conditions that account for a place’s mortality levels are rarely changed over short intervals of time and, instead, are experienced by multiple birth cohorts. Thus, in most cases, the levels of adult mortality in a place at time *t* reflect conditions that influenced mortality levels some time before, *t* − *k*, when adults born in the place were children. Since adult and child mortality are correlated, adult mortality disparities by place of residence at time t will also reflect child mortality disparities at time *t* − *k*. Because children born in places with higher mortality are also more likely to experience adverse early childhood conditions, it follows that adult migrants born in places with high mortality at time *t* − *k* are more likely to experience adult delayed effects in places of destination than migrants to the same place but born in places with lower mortality. Thus, if delayed effects are at all important, places that receive migrants who are disproportionately drawn from higher mortality places, will experience higher adult mortality, irrespective of conditions at destination. By the same token, migrants to a destination who are born in high mortality places should have higher adult mortality than migrants to the same destination but born in low mortality places.

The linkage between early exposures and adult health conjectured by DOHaD leads to the expectation that adult delayed effects will not be immediately offset by the healthy migrant effect experienced at destination. Delayed effects will only worsen adult migrants’ health and be reflected in a place of destination’s mortality level some time after migrants attain critical ages, e.g., past 40 or 50. Thus, if delayed effects exist, the age and origin composition of migrants arriving to a place could in part account for its adult mortality level, offsetting the healthy migrant effect. By the same token, when the salmon bias is significant, adult migrants who return to their place of origin may do so as a reaction to the manifestation of adult delayed effects. Consequently, the magnitude of the attenuation of the healthy migrant effect by delayed effects will be inversely related to the magnitude of the return migration flow induced by the salmon bias.

In sum, health selection, early experiences, and contemporaneous factors in places of residence are entangled and their influences hopelessly confounded by standard measures of mortality disparities by place of residence. These actually reflect the operation of multiple determinants, including those associated with a place’s health conditions, the composition of immigrants by age and place of birth (healthy migrant and DOHaD type of effects), the composition of the place’s outmigrants by age and health status (healthy migrant and DOHaD effects), and the composition by age and place of destination of returning migrants (salmon bias and DOHaD effects).

## Methods

### Mortality Data

The mortality microdata used in this study were retrieved from vital statistics records maintained by the Spanish National Institute of Statistics (INE) and cover the period between January 1, 2003 and December 31, 2019. All-cause mortality^5^ daily counts were aggregated by sex, single year age groups, and geographic location of birth and residence. We focus on the adult population aged 50 and over in 2003 and exclude the foreign born or those with missing information on place of birth. To preserve confidentiality, individuals whose place of residence was a municipality with less than 10,000 inhabitants are assigned the province of residence. Therefore our analyses can only be conducted at the provincial (or at a higher aggregation) level. In this paper we consider provincial information aggregated into 17 Autonomous Communities (AC) (regional NUTS-2 divisions).^6^ To facilitate presentation of results, some of these communities were merged together based on geographic proximity and demographic similarities. In all, we consider 11 regions. Figure S1 identifies the geographic location of the 11 AC and Table S1 displays their full names, acronyms, and the relation between the original 17 NUTS-2 divisions and the final 11 regions we consider in the paper. In addition, Table S1 also displays life expectancy at age 50 for males and females and estimated GDP and unemployment rate as of 2003, the initial year of the mortality data we use in the paper. The poorest AC’s, and also the ones exhibiting some of the lowest life expectancy, are Andalusia (AN) located in Southern Spain, Extremadura, Castilla-La Mancha and Murcia (ECMM), located in Spain’s central region, Asturias and Cantabria (ASC) in the North, and Canary Island, the most isolated Spanish region. In contrast, Madrid (MD) and Spain’s northernmost AC’s (PVNR) near the Spain-France border, are the richest and experience the highest life expectancy. Castilla and Leon (CL) and Galicia (GA) are outliers in the sense that, despite being among the poorest AC’s, they experience some of the lowest mortality levels.

### Population Data

Population data are publicly available as a series of microdata files for the period 2003–2020. They were retrieved from the so-called Continuous Register Statistics (*Padrón Continuo*). Individuals’ registration in these registers is compulsory by law for all Spanish residents and coverage is virtually complete. As all administrative registers, there might be lags in the registration of residential mobility. In addition, in the case of Spain, it also under reports movements of international migrants within the Spanish territory but this will not affect the analysis we carry out in this paper. Since many of these registration lags involve residential movements within the same municipality, especially in large urban areas, they cannot affect completeness of population counts nor do they influence our estimates.^7^

### Building Life Tables from Administrative Data

The main difficulty we face is that individual population records are not linked to individual death records. As a consequence, we cannot follow individuals residential histories and construct cohort life tables by place of birth, place of residence, and duration of residence, as one would with longitudinal data. However, as we show below, it is possible to construct pooled, pseudo-cohort life tables, by place of birth and place of residence.

**Fig. 1:**
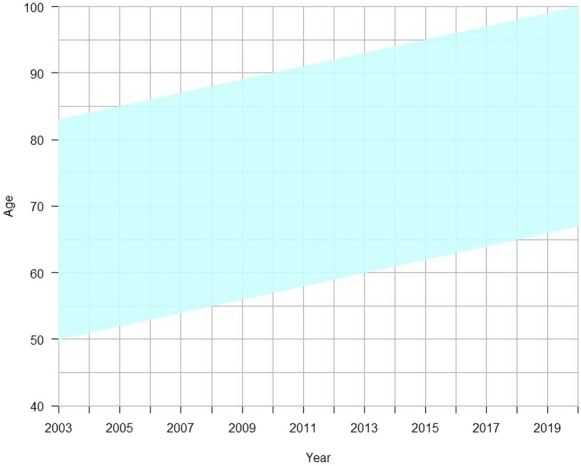
Lexis diagram showing the cohort built for this study

The Lexis diagram in Fig. 1 is a representation of the data we employ. The youngest cohort corresponds to those aged 50 on January 1st, 2003 and the oldest to those aged 82 in the same year. Each of these cohorts, as well as those between them, spans a total of 17 years and contributes to population exposure and mortality counts for age groups contained between the age interval (*x*, *x* + 1) at the outset (2003) to (*x* + 16, *x* + 17) at the end of the period under observation (2019).^8^ Importantly, the Lexis diagram shows that most cohorts contribute to mortality experiences to the same age group as do other cohorts but during different time periods (see below). Our aim is to construct a pooled life table for each place of birth and residence combinations that synthesizes the mortality experience during the entire period under observation. We will thus ignore period (or cohort) effects and focus only the pooled estimates.^9^

Consider a region of birth *B* and a region of residence *R* (*B* and *R* could be the same). For every place of birth $B$, and place of residence $R$, we build life tables starting at age 50. As described in Supplementary Materials, we adjust the number of deaths (and thus the corresponding conditional probabilities of dying) to avoid potential biases due to migration inflows and outflows during year t in a given place. For every year, age and sex, we compute the probability of dying ${q}_{R,B}^{\mathrm{^{\prime}}}\left(x,s\right)$ with the corrected number of deaths:

1 $$\begin{array}{c}{q}_{R,B}^{\mathrm{^{\prime}}}\left(x,s,t\right)=\frac{{{D}{\prime}}_{R,B}\left(x,s,t\right)}{{P{\prime}}_{R,B}\left(x,s,t\right)}\end{array}$$

For most cells in the 11 × 11 matrix of places of birth and residence and for a given one-year age interval, there is more than one ${q}_{R,B}^{\mathrm{^{\prime}}}\left(x,s,t\right)$ because different birth cohorts contribute to them at different times. To combine these into a pooled estimate of the corresponding conditional probability, weigh each ${q}_{R,B}^{\mathrm{^{\prime}}}\left(x,s,t\right)$’s by the fractional contribution to the total population exposed associated with each of the cohorts that contributes to the conditional probabilities, ${P}_{R,B}\left(x,s,t\right)/\sum_{t}{P}_{R,B}\left(x,s,t\right)$ thus yielding a pooled estimate:

2 $$\begin{array}{c}{q}_{R,B}\left(x,s\right)=\frac{\sum_{t}{q{\prime}}_{R,B}\left(x,s,t\right)*{P}_{R,B}\left(x,s,t\right)}{\sum_{t}{P}_{R,B}\left(x,s,t\right)}\end{array}$$

Out of a total 11,858 $R, B, x, s$ combinations (49 ages, 2 sexes, and 121 place of birth and residence combinations), there are 56 (0.5 percent) cases for which ${P}_{R,B}\left(x,s,t\right)=0$, mostly at the last age of the life table. In those cases, we fit a Gompertz curve and obtain an estimate of ${q}_{R,B}\left(x,s\right)$ at the missing ages. From those death probabilities from (2), we can obtain the corresponding mortality rates:

3 $$\begin{array}{c}{m}_{R,B}\left(x,s\right)=\frac{2\cdot {q}_{R,B}\left(x,s\right)}{2-{q}_{R,B}\left(x,s\right)}\end{array}$$

Thus, we will have a total of 242 (pooled) life tables for two sexes and 121 places of birth and residence combinations or, equivalently, 242 observed values of life expectancies at age 50, e_50_, the main dependent variable in our analysis. In addition, and for graphical purposes, we computed standardized mortality rates by place of birth and residence: for those, we employed the mortality rates from (3). They were thus computed as:

4 $$\begin{array}{*{20}c} {{\text{SMR}}_{{R,B}} = \sum\limits_{{\forall x}} {m_{{R,B}} } \left( {x,M} \right) \times \pi \left( {x,M} \right) + \sum\limits_{{\forall x}} {m_{{R,B}} } \left( {x,F} \right) \times \pi \left( {x,F} \right)} \\ \end{array}$$

where ${m}_{R,B}\left(x,M\right)$ and ${m}_{R,B}\left(x,F\right)$ are the male and female mortality rates for those residing in *R* who were born in *B* (from Eq. (3)) and $\pi (x,s)$ is the proportion of individuals of age *x* and sex *s* in a standard population.^10^

### Model for Life Expectancy at Age 50

**Fig. 2:**
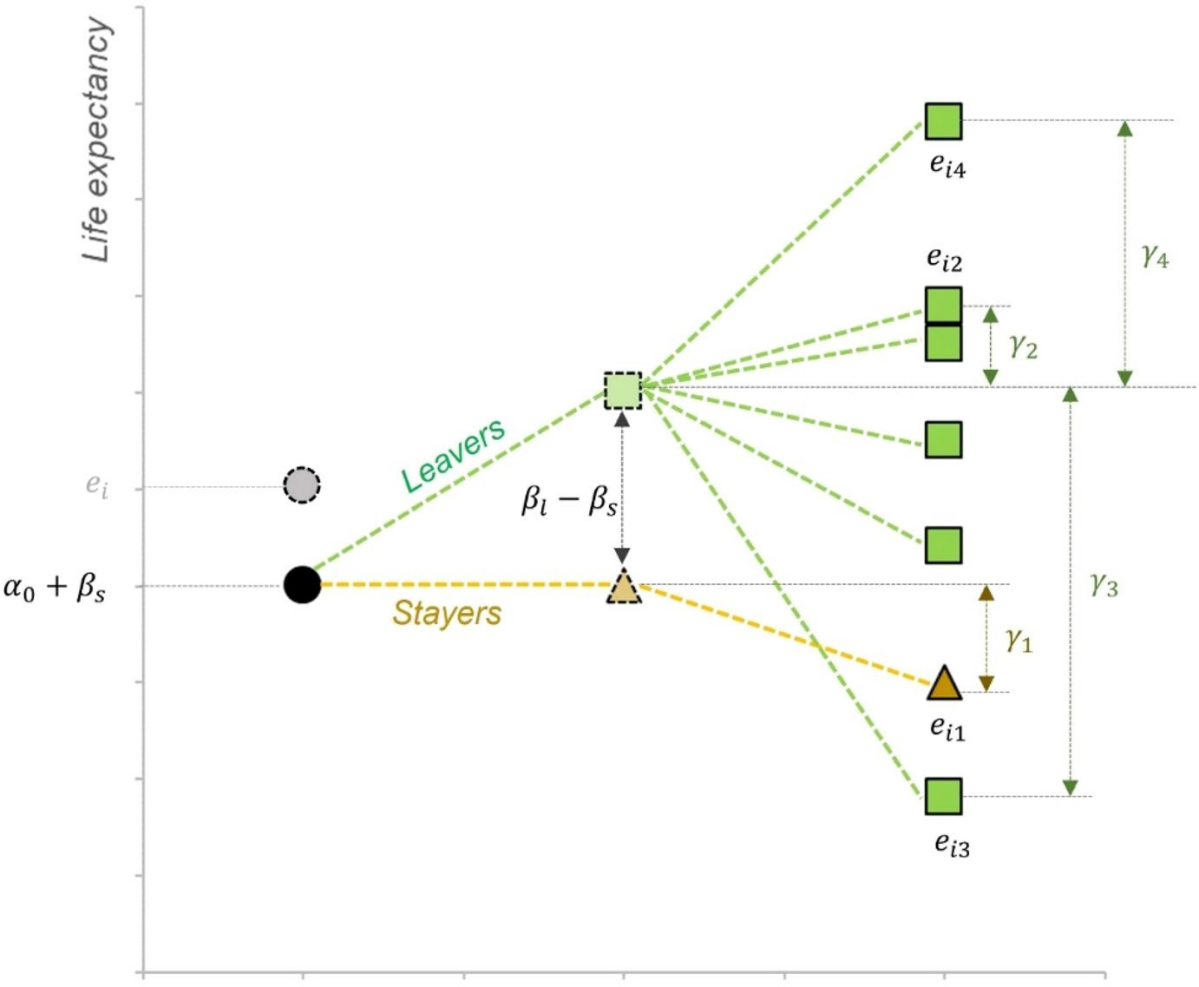
Diagram of the decomposition of life expectancy. The life expectancy ${e}_{i}$ (${e}_{50(i)}$) of those born in *i* is represented by the gray circle. The black circle (${\alpha }_{0}+{\beta }_{s}$) shows the baseline life expectancy plus the effect of place of birth for stayers. Movers or leavers experience a selection equal to (${\beta }_{l}-{\beta }_{s}$). The dotted triangles and squares are the life expectancy of stayers and movers, respectively, with no environmental effects. Environmental effects (${\gamma }_{j}$) are shown for each region of residence *j*. The weighted mean of all the life expectancies ${e}_{ij}$ on the right is equivalent to ${e}_{i}$, represented by the gray circle. (Color figure online)

We classify individuals according to their region of birth, *i*, and residence, *j*. When *i* = *j* we will refer to them as “stayers” and “leavers” otherwise. We propose a simple model for the observed life expectancy at age 50 in Spain as a function of combinations of places of birth and residence:

5 $$\begin{array}{c}{e}_{50}={\alpha }_{0}+\sum_{i=1}^{r}\left({p}_{ii}\times {\beta }_{si}+\left({p}_{bi}-{p}_{ii}\right)\times {\beta }_{li}\right)+\sum_{j=1}^{r}{p}_{rj}\times {\gamma }_{j}+\varepsilon \end{array}$$

where ${\alpha }_{0}$ is a constant, ${p}_{ij}$ is the probability that an individual is born in *i* and resides in *j*, ${p}_{bi}$ is the probability of being born in *i*, ${p}_{rj}$ is the probability of residing in *j*; ${\beta }_{si}$ is a coefficient that reflects the effects of place of birth on mortality of stayers in *i*, ${\beta }_{li}$ is a coefficient for the effects of place of birth on mortality of leavers from *i*, ${\gamma }_{j}$ is the effect on mortality place of residence *j* irrespective of place of birth and, finally, $\varepsilon$ is an idiosyncratic error. The term $\left({p}_{bi}-{p}_{ii}\right)$ equals the proportion of individuals who are born in *i* and reside elsewhere. Under some conditions spelled below, the difference ${({\beta }_{li}-\beta }_{si})$, is a measure, albeit imperfect, of health selection of migrants from *i*. A graphic rendition of expression (5) is in Fig. 2.^11^

Proper interpretation of parameters in model (3) requires some assumptions. First, because ${\beta }_{li}$ and ${\beta }_{si}$ depend only on place of birth, *i*, and not on place of residence, *j*, their difference can be interpreted as a measure of health selection of leavers from *i* (relative to stayers), averaged out across places of destination. Second, note that the estimate of the parameter reflecting effects of a place of residence, ${\gamma }_{j}$, does not depend on *i* and it is assumed to be the same for all migrants arriving in *j*, irrespective of their place of birth. Underlying expression (5), there is a simple model for the life expectancy of individuals who were born in place *i* and migrated to place *j*, namely,

6 $$\begin{array}{c}{e}_{50(i,j)}={\alpha }_{0}+{\beta }_{si}\times {S}_{i}+{\beta }_{li}\times {L}_{i}+{\gamma }_{j}\times {R}_{j}\end{array}$$

where ${e}_{50(i,j)}$ is the life expectancy at age 50 of those born in *i* and reside in *j*, ${S}_{i}$ is a binary 0/1 dummy variable set to 1 for stayers born in *i*, ${L}_{i}$ is a dummy variable set to 1 for leavers born in *i*; ${R}_{j}$ is a dummy variable equal to 1 when the place of residence is *j*. The coefficients ${\alpha }_{0}$, ${\beta }_{si}$, ${\beta }_{li}$ and ${\gamma }_{j}$ have the same interpretation as in (3). We seek to identify 3*r* + 1 parameters, namely, a constant, ${\alpha }_{0}$, parameters ${\beta }_{si}$ and ${\beta }_{li}$ for r regions of birth and parameter $\gamma$ for r regions of residence. We have a total of $r\times r$ equations for values of life expectancy of all possible pairs of indexes for places of birth and residence, *i* and *j*. The parameters of (6) were estimated using a simple linear model.^12^

### Heritability of Life Expectancy

The parameter estimates from model 6, can be used to compute a quantity of great interest, namely, the “heritability” of mortality conditions associated with places of birth. Our previous discussion suggests that migrants carry with them risks associated with conditions experienced during early childhood in the place of birth that can manifest as adult delayed effects, irrespective of region of destination. We propose an estimator of heritability that includes components of total variance of life expectancy across regions associated with place of residence and place of birth. The first component is a function of place of birth and we refer to it as the “inherited” component, I:

7 $$\begin{array}{c}I=\sum_{i=1}^{r}\left({p}_{ii}\times {\beta }_{si}+\left({p}_{bi}-{p}_{ii}\right)\times {\beta }_{li}\right)\end{array}$$

In this expression, the contribution of stayers and leavers is different as the latter is modified by health selection (if any). The second component is a function of place of residence and we refer to it as the “environment” component, E:

8 $$\begin{array}{c}E=\sum_{i=1}^{r}{p}_{rj}\times {\gamma }_{j}\end{array}$$

Following the terminology in population genetics, we will define heritability of health conditions, ${h}^{2}$, as the fraction of the total variance in life expectancy at age 50 (the “phenotype”) explained by place of birth (“additive allelic variance”) and, correspondingly, the effect of environment is the fraction of the total variance in life expectancy at age 50 explained by place of residence (“environmental effects”).

9 $$\begin{array}{c}{h}^{2}=\frac{\mathrm{Var}\left(I\right)}{\mathrm{Var}\left(E\right)+\mathrm{Var}\left(I\right)}\end{array}$$

with $${h}^{2}\in [\mathrm{0,1}]$$.

Using Eqs. (7), (8) and (9), we can express ${h}^{2}$ as

10 $$\begin{array}{c}{h}^{2}=\frac{{\sum }_{i\in \left[1,r\right]}\left[{p}_{ii}\times \left(1-{p}_{ii}\right)\times {\beta }_{si}^{2}+{\overline{p}}_{i}\times \left(1-{\overline{p}}_{i}\right)\times {\beta }_{li}^{2}\right]}{{\sum }_{i\in \left[1,r\right]}\left[{p}_{ii}\times \left(1-{p}_{ii}\right)\times {\beta }_{si}^{2}+{\overline{p}}_{i}\times \left(1-{\overline{p}}_{i}\right)\times {\beta }_{li}^{2}+{p}_{ri}\times \left(1-{p}_{ri}\right)\times {\gamma }_{i}^{2}\right]}\end{array}$$

where

11 $$\begin{array}{c}{\overline{p}}_{i}={p}_{bi}-{p}_{ii}\end{array}$$

Higher values of ${h}^{2}$ correspond to higher influence of region of birth conditions (*i.e.*, the effects of early conditions that manifest as adult delayed conditions are strongly inherited). Conversely, smaller values of ${h}^{2}$ will obtain when mortality heterogeneity is mostly associated with characteristics of places where individuals reside at the time of death.^13^

## Results

Table 1 displays basic counts of population and deaths by region of birth. The cohorts include 12.375 million individuals, 4.758 million of which (38.4 %) died within the period of observation.

**Table 1:** Descriptive statistics of the cohort

Birth region	Females		Males	
	Individuals *(thousands)*	Deaths *(thousands)*	Individuals *(thousands)*	Deaths *(thousands)*
Andalusia (AN)	1,469.6	516.5	1,289.6	580.6
Aragon (AR)	254.5	89.8	225.3	101.9
Asturias, Cantabria (ASC)	306.7	109.6	254.5	118.3
Castile and Leon (CL)	778.5	239.0	692.1	284.5
Canary Islands (CN)	209.0	69.2	182.7	79.2
Catalonia (CT)	559.8	192.1	493.4	216.9
Extremadura, Castilla-La Mancha, Murcia (ECMM)	1,130.9	384.6	1,003.7	436.9
Galicia (GA)	597.0	194.7	508.8	220.3
Madrid (MD)	356.5	108.6	310.0	127.0
Basque Country, Navarre, La Rioja (PVNR)	387.3	128.8	330.2	114.6
Valencia, Balearic Islands (VIB)	548.8	195.0	486.2	219.4
Total	6,598.5	2,227.8	5,776.7	2,529.7

Tables 2 and 3 display matrices of life expectancy at age 50 by region of birth and residence. Consider first life expectancy by place of birth. Among males, it ranges between 27.8 (born in Catalonia and living in Extremadura-Castilla-La Mancha-Murcia) and 32.7 years (born in Castile-and-Leon and living in Madrid). Among females, the range is between 33.8 (born in Valencia-Balearic Islands and living in Andalusia) and 37.6 years (born in Castile-and-Leon and living in Madrid). The highest life expectancy by region of birth is for those born in Castile-and-Leon (31.5 years for males and 37.0 for females) whereas the lowest are among those born in Andalusia for males (29.3 years) and Canary Islands for females (34.6 years). Regions that follow closely the advantageous performance of Castile-and-Leon are Aragon, Galicia or Madrid. Instead, populations born in Canary Islands and Valencia-Balearic Islands are close to the region with the poorest performance.

**Table 2:** Life expectancy of males at age 50

Resid.	AN	AR	ASC	CL	CN	CT	ECMM	GA	MD	PVNR	VIB	All regions
Birth												
AN	*29.0*	*29.0*	*28.5*	***30.6***	29.9	*29.7*	*29.7*	30.1	**31.0**	*29.6*	*29.3*	**29.3**
AR	*29.8*	***30.6***	*29.8*	***31.1***	***30.8***	***31.3***	*29.8*	***30.9***	***32.2***	***31.3***	***30.8***	**30.8**
ASC	*29.3*	*29.2*	*29.4*	30.2	***31.0***	*29.8*	29.9	30.1	***31.8***	*29.8*	30.1	**29.6**
CL	***30.8***	***31.5***	***30.7***	***31.2***	***32.1***	***31.7***	***31.2***	***31.2***	***32.7***	***31.3***	***30.5***	**31.5**
CN	*28.4*	***31.8***	***31.1***	***30.6***	*29.3*	30.2	30.2	***30.8***	***31.9***	*29.6*	***30.9***	**29.3**
CT	*28.3*	*29.7*	*28.9*	*29.7*	30.3	***30.4***	*27.8*	*29.3*	***31.4***	30.3	*29.3*	**30.4**
ECMM	*29.6*	30.2	*29.6*	***30.9***	***30.6***	30.2	30.3	*29.8*	***31.6***	30.1	30.0	**30.5**
GA	***30.7***	*28.8*	*29.5*	***30.5***	***31.0***	***31.4***	***30.8***	30.2	***32.3***	***30.7***	***30.5***	**30.3**
MD	*29.1*	*29.3*	*29.6*	***30.8***	*29.8*	29.9	*29.4*	30.0	30.3	***30.8***	*29.1*	**30.2**
PVNR	*28.5*	*29.8*	*29.3*	***30.6***	***31.1***	30.3	*29.8*	*29.5*	***31.6***	30.2	*29.6*	**30.3**
VIB	*29.2*	30.0	***30.7***	***32.2***	*29.4*	30.3	*29.7*	***30.9***	***30.9***	***30.6***	*29.7*	**29.8**
All regions	**29.1**	**30.5**	**29.5**	**31.1**	**29.4**	**30.3**	**30.2**	**30.2**	**31.3**	**30.4**	**29.7**	**30.1**

The columns indicate place of residence and the rows indicate place of birth. The last column is for life expectancy of males born in each region regardless of residence whereas the last row is life expectancy of males residing in each region regardless of place of birth. Bold italic (italic) color indicates a life expectancy that is at least 0.3 years over (below) the national average

**Table 3:** Life expectancy of females at age 50

Resid.	AN	AR	ASC	CL	CN	CT	ECMM	GA	MD	PVNR	VIB	All regions
Birth												
AN	*34.3*	*34.9*	*34.9*	*35.4*	35.6	35.5	*34.6*	*35.3*	***36.3***	35.5	*34.9*	**34.7**
AR	*34.5*	***36.1***	35.5	***36.2***	***37.3***	***36.7***	***36.1***	***37.0***	***37.0***	***36.9***	***36.0***	**36.2**
ASC	*35.1*	35.9	35.8	***36.3***	***37.5***	***36.1***	***36.0***	*35.4*	***36.8***	***36.2***	35.5	**35.9**
CL	35.9	***36.6***	***36.4***	***36.8***	***37.5***	***37.1***	***36.2***	***36.5***	***37.6***	***37.2***	***36.4***	**37.0**
CN	*33.9*	***37.1***	35.5	35.7	*34.6*	*34.7*	***36.1***	***34.7***	***35.3***	***35.4***	***34.3***	**34.6**
CT	*34.2*	*35.1*	35.6	35.9	*35.3*	35.9	*34.5*	35.6	***36.5***	***36.4***	*34.9*	**35.8**
ECMM	*34.6*	35.9	35.7	***36.2***	***36.1***	35.8	*35.3*	*35.1*	***36.8***	***36.3***	*35.1*	**35.7**
GA	35.6	*34.8*	***36.1***	***36.2***	***36.9***	***36.8***	35.6	***36.0***	***37.3***	***36.7***	35.9	**36.1**
MD	*34.5*	*35.4*	35.5	*35.4*	35.8	35.9	*35.1*	35.5	***36.2***	***36****.6*	*35.1*	**36.0**
PVNR	*34.8*	35.7	***36.0***	***36.2***	***36.1***	***36.2***	*35.1*	*34.9*	***36.7***	***36.3***	35.6	**36.3**
VIB	*33.8*	*35.2*	***36.0***	***36.3***	***37.0***	35.8	*34.9*	*35.1*	***36.3***	***36.0***	*35.0*	**35.0**
All regions	**34.3**	**36.0**	**35.8**	**36.7**	**34.7**	**35.9**	**35.3**	**36.0**	**36.7**	**36.5**	**35.1**	**35.7**

The columns indicate place of residence and the rows indicate place of birth. The last column is for life expectancy of females born in each region regardless of residence whereas the last row is life expectancy of females residing in each region regardless of place of birth. Bold italic (italic) color indicates a life expectancy that is at least 0.3 years over (below) the national average

Turning now to life expectancy by place of residence, we observe that the highest life expectancy is among those residing in Madrid (31.3 years for males and 36.7 for females), whereas those living in Andalusia have the lowest (29.1 years for males and 34.3 for females). Note that life expectancy among those living in Basque Country-Navarre-La Rioja or Castile-and-Leon is high, regardless of their place of birth, whereas for those living in Valencia-Balearic Islands are almost always among the lowest, regardless of place of birth.

The range of variation of estimates of life expectancy by place of birth and by place of residence are similar to each other. In addition, life expectancy by place of birth and by place of residence are correlated (${R}^{2}\,$=0.67, *Spearman* = 0.75 for men and ${R}^{2}\,$=0.86, *Spearman* = 0.90 for women). The association is tight because a significant fraction of the population resides in the place of birth (about 72% of Spain’s population). However, and specially for males, the mapping of one on the other is far from perfect.

A final observation is that, as expected by the healthy migrant conjecture, life expectancy of stayers is lower than that of leavers for all regions of birth, except Madrid and Catalonia (and, among females only, in Basque Country-Navarre-La Rioja).

**Fig. 3:**
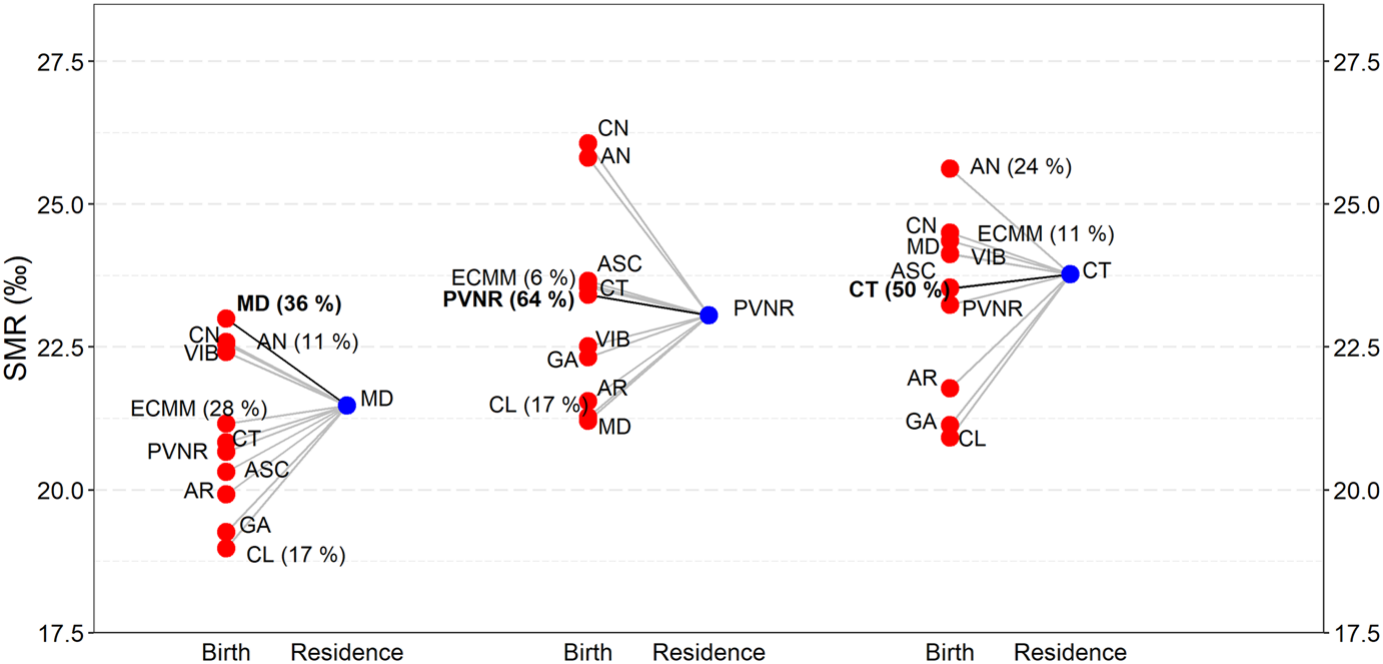
Composition of annual SMR (‰) in three regions of residence by origins. Blue circles are annual SMR of residents in Catalonia (left), Basque Country-Navarre-La Rioja (center) and Madrid (right). Red circles are SMRs of those born in a region and residing in Catalonia, Basque Country-Navarre-La Rioja or Madrid, respectively. The fraction of residents in a region that were born in another is displayed when it exceeds 5%. (Color figure online)

**Fig. 4:**
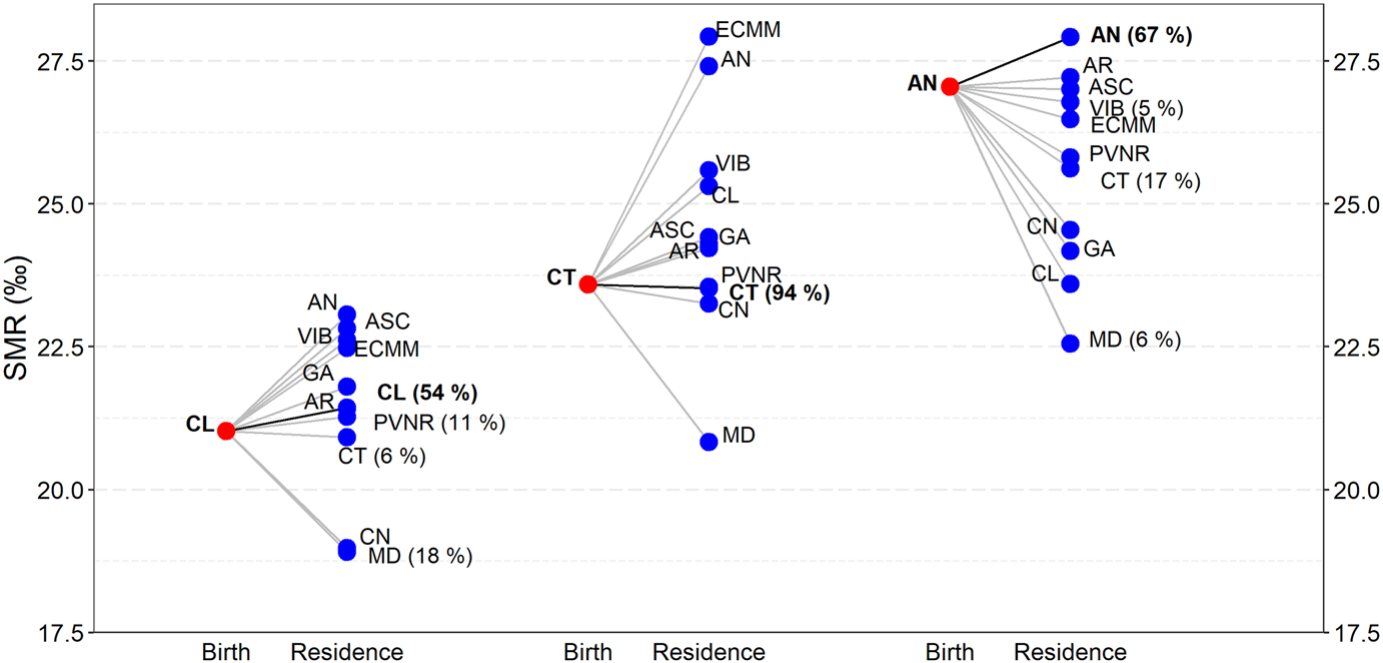
Composition of annual SMRs (‰) in three regions of birth by destinations. Red circles are SMRs of natives of Castile-and-Leon (left), Catalonia (center) and Andalusia (right). Blue circles SMRs of those residing in a certain region and born in Castile-and-Leon, Catalonia or Andalusia, respectively. The fraction of residents in a region that were born in another is displayed when it exceeds 5%. (Color figure online)

Figure 3,^14^ shows the influence of migrants on the observed mortality rate in places of destination or residence. Madrid stands out as the place where immigrants born in all other regions have lower mortality rates than individuals born there. This is a case in which migrants to Madrid increase the region’s life expectancy. Though to a different degree, the same applies to regions Asturias-Cantabria, Andalusia, Galicia, Canary Islands, Valencia-Balearic Islands and Basque Country-Navarre-La Rioja. Conversely, immigrants to Castile-and-Leon and Aragon experience higher mortality rates than those born in these regions and contribute to lower their life expectancy. The same occurs in Catalonia and Extremadura-Castilla-La Mancha-Murcia, though, in these cases, the phenomenon is mostly due to the high fraction of individuals born in Andalusia among migrants. Note that, with the exception of Madrid and Catalonia, mortality is higher among stayers in a place than among leavers out of the place (Fig. 4). Andalusia is an extreme case, where stayers have the highest mortality rate among all regions of residence.

The lowest variance of mortality rates by place of birth corresponds to residents in Castile-and-Leon, Galicia and Madrid, which stand out among the regions with lower mortality by residence. In general, places where residents have lower mortality levels also have lower variance of mortality rates across all origins of residents (${R}^{2}\,$ = 0.18, *Spearman* = 0.41). This suggest the operation of a few mechanisms: either there is a strong convergence of mortality of migrants, e.g., a powerful influence of environments at destination, or tight similarity of mortality levels among migrants due to place of birth influences or, finally, similar magnitude and nature of health selection.

Finally, the lowest variability of mortality across places of residence is found among those born in Castile-and-Leon or Madrid, whereas the highest is among those born in Canary Islands, Catalonia or Valencia-Balearic Islands. Overall, mortality rates by place of birth are positively correlated with the variance of mortality rates across places of residence (${R}^{2}\,$ = 0.33, *Spearman* = 0.74). This could be due to the fact that those born in places with lower mortality tend to be less affected by levels of mortality in the place of destination.

### Estimates of Parameters for Life Expectancy

The linear model in Eq. 6 fits the data well with ${R}^{2}\,$= 0.72 for males and ${R}^{2}\,$ = 0.77 for females. The baseline life expectancy at age 50 (constant plus ${\beta }_{si}$) corresponds to the contribution of place of birth to stayers (columns 1 for females and 4 for males). It hovers around a mean of ${\alpha }_{0}\,$ =3 0.0 years for males and ${\alpha }_{0}\,$=35.7 years for females, a six year difference (Table 4).The highest contribution by place of birth is for individuals born in Aragon, Extremadura-Castilla-La Mancha-Murcia and Castile-and-Leon among males, and Castile-and-Leon and Galicia among females. Differences between regions are about 2.1 years for men (Aragon and Madrid) and 2.6 years for women (Castile-and-Leon and Canary Islands).

**Table 4:** Decomposition of life expectancy

	Female			Male		
	Baseline	Selection	Residence	Baseline	Selection	Residence
AN	35.5	− 0.3	− 1.2	30.0	− 0.4	− 1.0
AR	36.2	0.1	− 0.1	30.9	− 0.2	− 0.3
ASC	35.8	0.2	− 0.1	30.0	0.1	− 0.5
CL	36.5	0.3	0.3	30.6	0.8	0.5
CN	33.9	1.4	0.7	28.9	1.6	0.4
CT	35.7	− 0.2	0.2	30.3	− 0.8	0.1
ECMM	35.8	0.0	− 0.4	30.7	− 0.5	− 0.5
GA	36.3	− 0.2	− 0.3	30.1	0.5	0.0
MD	35.3	0.2	0.8	28.8	1.1	1.4
PVNR	35.8	0.0	0.5	30.1	− 0.1	0.1
VIB	33.5	0.1	− 0.5	30.0	0.4	− 0.3

*e50(ij)* = *α*0 + *βsi* + *βli* + *γj* where *α*0 + *βsi* is the baseline, *βli − βsi* is the selection term for “leavers”, and *γj* is the residence term

Columns 2 for females and 5 for males display the contribution of leavers’ selection effects (${\beta }_{li}\hspace{0.17em}-\hspace{0.17em}{\beta }_{si}$). These values are mostly positive and the largest selection effect, about 1.4 years for females and 1.6 for males, are experienced by those who leave Canary Islands. Conversely, outmigrants from some regions, mainly Andalusia and Catalonia, appear to be negatively selected compared to stayers. Overall, the size of these selection effects is larger among males than among female, as are their variances (0.54 for men and 0.21 for females).

Finally, estimates of place of residence’s effects ${\gamma }_{j}$, varies substantially across regions of residence. The largest values are for Madrid, where the residential advantage is equivalent to 1.4 years for males and 0.8 years for females. In contrast, some regions have a negative impact as in Andalusia where there are reductions of 1.0 years 1.2 years for males and females, respectively.

### Income, Migration and Life Expectancy

The association between GDP per capita of regions and estimates of residence effects is tight (${R}^{2}$ = 0.5), confirming that environments of a place matters. This suggests that those residing in Madrid, Castile-and-Leon, Basque Country-Navarre-La Rioja or Catalonia have higher life expectancy, partly because they have higher incomes or enjoy health benefits of improved infrastructure. Canary Islands stands out as an exception, as it is a below- average-income region with high life expectancy among those who migrate to it. Thus, immigrants to Canary Islands have higher life expectancy (over 30.6 years for men and over 36.4 years for women) than do Canary Islands’ stayers (29.3 years for men and 34.6 for women). This might be a result of the fact that migrants to Canary Islands are a highly selected population (relative to stayers in Canary Islands), much less affected by adverse conditions than Canary Islands’ stayers. It is likely that a high fraction of migrants to Canary Islands are skilled workers and professionals who take jobs that natives cannot. Also, it is known that Canary Islands is a favorite destination place for mainland’s high income groups who settle in Canary Islands right before and after retirement. In both cases, the health status of migrants to Canary Islands should be superior to those who are born in the region.

### Heritability of Life Expectancy

The heritability coefficient of life expectancy in Spain is ${h}^{2}\,$ = 0.42 for males and ${h}^{2}\,$= 0.43 for females. This means that an important fraction of the variability of life expectancy at age 50 is explained by conditions that individuals take with them when they migrate to another region. We argue that these conditions reflect experiences in places of birth that have delayed adult effects. This estimate of heritability is a lower bound, as it does not reflect the impact of salmon bias, that is, the effects of place of birth on individuals who returned to them because of poor health possibly induced by adult delayed effects.

## Discussion

A rigorous accounting of factors that might explain regional mortality disparities requires, at the very least, to distinguish between effects associated with place of birth and place of residence. Not only does this information help us discern more precisely the impacts of healthy migrant and salmon bias effects, but it also offers a viable strategy to assess the magnitude of adult delayed effects. Admittedly, considering only place of birth and residence in 2003 is not an optimal strategy to assess health selection or test DOHaD hypotheses. However, except in populations with only weak regional mobility, mortality estimates by place of birth and residence reveal much more than standard measures of mortality.

Our results lead to a number of inferences. First, as showed in the case of the USA (Fletcher et al., 2023; Xu et al., 2020), separating the contribution of places of birth and places of residence, identifies important heterogeneity in mortality disparities. The differentials in life expectancy at age 50 by region of residence (2.4 years for women, 2.2 years for men) are of the same order of magnitude as those observed by region of birth (2.4 years for women, 2.2 years for men). The size of these differences is not trivial. In fact, in Spain at least, they are comparable to mortality differentials by education: the difference between those with primary education and those with college education or more are of the order of 1.5 years for females and 2.5 for males (Requena, 2017).

Second, we find some support for healthy migrant effects. With the exception of those who move to places with the highest levels of mortality (Andalusia, Canary Islands, Asturias-Cantabria), leavers from all regions tend to experience lower mortality than counterparts who stay in their places of birth. There are two extreme illustration: (i) Madrid stands out as the destination for leavers with the highest life expectancy. Indeed, out-migrants to Madrid originating in 8 out of 10 regions experience highest life expectancy as Madrid residents. This could be a result of better conditions in Madrid, the outcome of particularly strong positive selection or both; (ii) conversely, out-migrants to Andalusia originating in 8 out of 10 regions experience the lowest life expectancy among all possible destinations. This could be due to strong negative health selection, poor environmental conditions in Andalusia or both. One could argue that the net differences between life expectancy of leavers and stayers reflect both the impact of places of destinations’ environment and the effects of selection (negative or positive). However, the fact that, with the exception of Andalusia, Canary Islands and Asturias-Cantabria, the bulk of differences always favors leavers over stayers, suggest that selection might be playing the dominant role.

Third, we find strong evidence suggesting the importance of place of birth as an explanatory factor of regional mortality disparities. First, out-migrants from places with low stayers’ mortality experience the lowest variance in mortality levels and, in addition, these are very similar to mortality levels in their places of birth, irrespective of destination. This strongly suggest that out-migrants inherit conditions associated within their place of birth or, equivalently, are less influenced by a place of destination’s environment. This finding is confirmed by the high value of Spain’s coefficient of heritability which indicates that between 42 and 43% of the total variance of life expectancy at age 50 is explained by place of birth.

Fourth, migrants can shape observed mortality in places of destination. Thus, observed regional mortality levels in Spain are associated with the composition of migrants by places of origin. For instance, immigrants to Catalonia increase its mortality level. This is largely explained by the fact that a high proportion of these migrants (47%, or 24% of all Catalonia residents) originate in Andalusia, the place with the least favorable mortality regime. If, instead, migrants originated in Castile-and-Leon, the annual standardized mortality rate would drop from 21.5 to 20.5 ‰. Migrants increase mortality levels in Castile-and-Leon and Aragon as well, but reduce it in all other places.

Finally, decomposition of life expectancy enables us to partially disentangle effects of health selection from residence effects. The value of the estimates is positive, as expected when there are healthy migrant effects. Health selection is highest among those born in Canary Islands, a geographically isolated region where successful outmigration could be more tightly constrained by health conditions, regardless of destination. Lastly, we find that, as expected, the strength of effects of place of residence is correlated with economic conditions of regions.

There are a number of shortcomings that limit the reach of our inferences. First and foremost, we ignore the role of leavers’ timing of migration and duration of residence in places of birth and destination. Thus, we cannot distinguish those who migrated right after birth from those who migrated just before retirement nor can we take into account repeated migration events. By the same token, because we are not working with migration histories, we cannot separate stayers who never moved from stayers who experience one or multiple migration events. Ultimately, place of birth is a highly imperfect indicator of strategic lifetime exposures, albeit one that is better than none.

Second, our estimates of healthy migrant effects are coarse because they are computed assuming that place of destination is unrelated to levels of health selection among immigrants to it. It is possible that health selection is more elevated among migrants to the most attractive regions, those where pressure on new arrivals exerted by labor markets competition are greater. Similar limitations apply to inferences about salmon bias.

Finally, two important issues we intend to pursue in future research. First, we can decompose differences in life expectancy by cause of death and age groups. Doing so offers a different source of empirical evidence to test the DOHaD conjecture. Second, rather than using Autonomous Communities, we could define lower level of aggregation and use a well-defined classification scheme to group provinces. Although we may lose some power in doing so, we will be able to perform analyses with higher granularity, including an assessment of variability of the heritability coefficient by place of origin.

## Conclusions

Following other research on the subject (Fletcher et al., 2023; Xu et al., 2020), the main goal of this paper is to argue that the study of regional mortality is enriched if one uses statistics computed by place of birth and place of residence. We showed that doing so leads to somewhat different assessments about the nature of regional disparities. In particular, disparities by place of birth are not the same as those by place of residence. This should matter considerably in populations that experience significant residential mobility. With help from some simplifying assumptions, we illustrate that these type of data leads to important insights about the nature of health selection of migrants to a place. Simultaneously, it is possible to compute estimates of effects of a place of residence that are more precise, e.g., less contaminated by the influence of migrants’ mortality on place of residence’s mortality. We propose an index of heritability of health-related conditions prevailing in places of birth among those who leave. The index reflects the importance of early exposures and their manifestation as delayed effects. This index could be an important source of new empirical evidence to test hypotheses abut adult delayed effects, one that is not available in standard studies of regional mortality disparities.

Finally, our findings for Spain are consistent with and comparable to those for the USA. This suggests the intriguing possibility that perhaps they reflect phenomena that are not unique or pathological but quite general in modern populations. If future research does indeed reveal that the regularities uncovered in the USA and Spain apply more generally, the findings will be an unexpected source of empirical evidence for DOHaD’s conjectures.

### Supplementary Information

Below is the link to the electronic supplementary material.

Supplementary file1 (PDF 538 kb)

## Footnotes

1 These gaps had been reduced to 3.3 years for provinces, but increased to 2.8 years for Autonomous Communities as of 2019, just before the Covid-19 epidemic. Moreover, this evolution between 2003 and 2019 depends largely on sex and age (INE, 2022).

2 The Developmental Origins of Health and Disease (DOHaD) is a well-developed theoretical framework that emerges from empirical investigations relating cohorts’ health and mortality across their lifetimes. Although its origins go back to studies in the 1920’s and 1930’s, the most important source is the work by Barker and colleagues at the University of Southampton (citations in text). Barker’s formulation has been augmented, enhanced and enriched principally by the work of Gluckman, Hanson and Bateson (citations in text) who proposed a number of conjectures about mechanisms linking early exposures to adult delayed outcomes, including those rooted in genetic and epigenetic modifications and adaptive ancestral constraints.

3 A handful of past studies has examined the impact of migration regional or small area disparities of mortality (Ocaña-Riola et al., 2009) and health (Polissar, 1980) but none of it is focuses on the mechanisms that interest us here.

4 We use data that does not contain information about timing of events. As a consequence, we cannot assess duration of exposures to risks associated with contextual factors in either place of residence or birth. Throughout, we will assume that most adult migrants spend their infancy and early childhood in their birthplaces or, equivalently, that only a small fraction of migrants leave their place of birth before attaining young adult ages.

5 The official death registration data gathered by the Spanish Vital Statistics assembled by the Instituto Nacional de Estadistica (INE) is in compliance with and uses the (International Classification of Diseases, 9th Revision (ICD-9) codes 001–799, and 10th Revision (ICD-10) codes A00-R99).

6 Due to their small size, we excluded populations born or residing in Ceuta and Melilla.

7 The Continuous Register was created to coordinate all the municipal registers in Spain. The term municipal register refers to an administrative registry where residents of a municipality are listed. Thus, the data from municipal registers constitutes proof of the individual’s residence in the municipality. Organization, maintenance, updates, and custody of the municipal registry is handled by town councils. To minimize duplication and facilitate coordination between data holders, these councils forward to INE monthly updates with changes in their registers. This avoids duplication and facilitates coordination between official data stakeholders.

8 Altogether, we have information for 12,375,155 individuals.

9 There are alternative strategies for combining the information available to compute the desired estimates. We include a description of these in Supplementary Materials. Two of the alternative strategies are suitable for producing estimates of period (or cohort) effects, if one so desires. Importantly, though, all of these strategies produce very similar **pooled** estimated life tables for the period under consideration.

10 We use the population of Spain on January 1st, 2020 as the standard.

11 To facilitate comparisons with results from other research on the same problem, we borrow the figure design from the work by Xu and coauthors (2020).

12 We estimated the model r times and in each case we used a different region as the omitted category for birth and residence. We then computed the mean of these model estimates, so that the *γ*_*j*’s are centered around 0. By doing so, we facilitate the interpretation of the selection term.

13 Had we used provinces as the smallest units of aggregation, we could compute estimates of heritability associated with each province. This would enable us to rank places according to heritability and assess its variability across places. The use of provinces, however, is a messy enterprise because it requires grouping rules to aggregate them in a meaningful way and avoid numerical problems caused by small number of death in some of them.

14 As is the case of Figs. 2, 3 and 4 are similar in design to those used for the analysis of US state mortality disparities (Xu et al., 2020).
