# Supplementary material for: Influence of Place of Birth on Adult Mortality: The Case of Spain
Source: Eur J Popul. 2023 Sep 7;39(1):30. doi: 10.1007/s10680-023-09679-y (PMC10484828; doi:10.1007/s10680-023-09679-y)

# Supplemental materials

Figure S1: Geographic units employed in the study

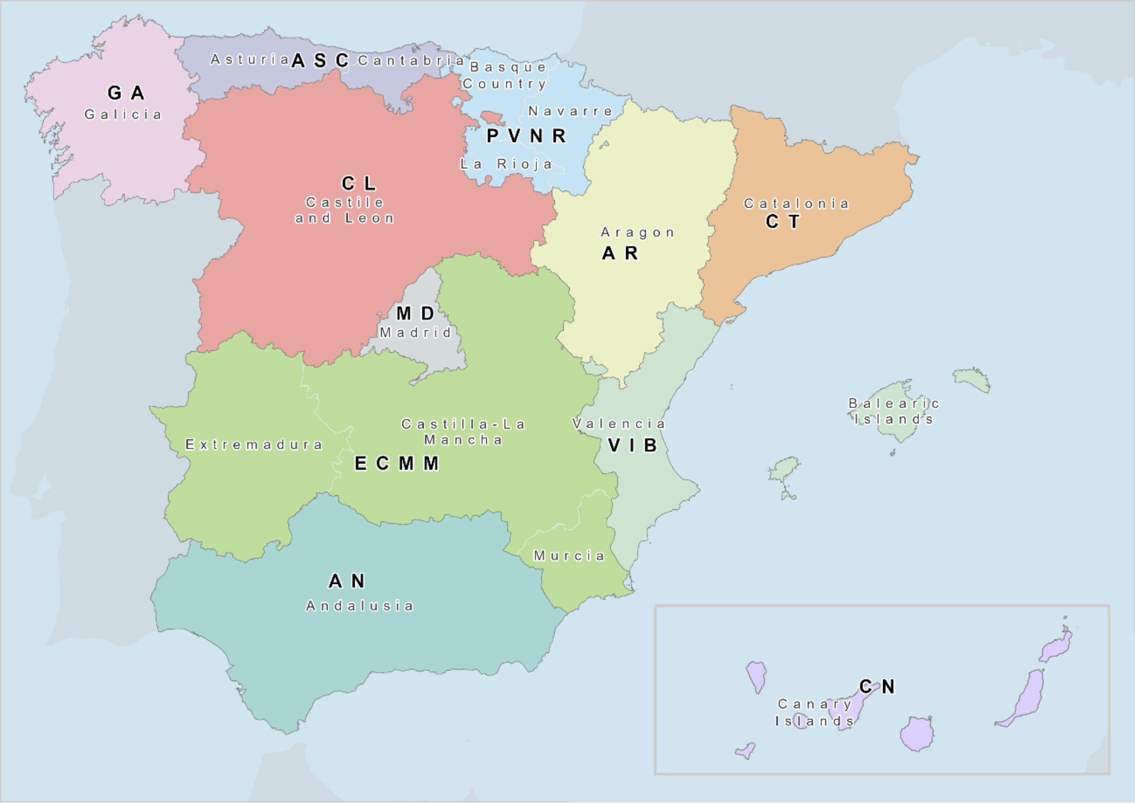

Table S1: Geographic units, life expectancy, GDP *per capita* (in euros) and unemployment rate

| **Unit** | **NUTS-2 regions** | $e_{50}^{m}$ | $e_{50}^{f}$ | **GDP *per capita*** | **Unemployment rate (%)** |
|---|---|---|---|---|---|
| AN | Andalusia | 29.1 | 34.3 | 14,537 | 19.0 |
| AR | Aragon | 30.5 | 36.0 | 20,282 | 7.4 |
| ASC | Asturias, Cantabria | 29.5 | 35.8 | 16,736 | 11.6 |
| CL | Castile and Leon | 31.1 | 36.7 | 17,363 | 11.4 |
| CN | Canary Islands | 29.4 | 34.7 | 18,206 | 10.7 |
| CT | Catalonia | 30.3 | 35.9 | 22,818 | 10.7 |
| ECMM | Extremadura, Castilla-La Mancha, Murcia | 30.2 | 35.3 | 14,732 | 13.4 |
| GA | Galicia | 30.2 | 36.0 | 15,215 | 12.9 |
| MD | Madrid | 31.3 | 36.7 | 25,053 | 7.8 |
| PVNR | Basque Country, Navarre, La Rioja | 30.4 | 36.5 | 23,030 | 8.6 |
| VIB | Valencia, Balearic Islands | 29.7 | 35.1 | 18,547 | 11.8 |
| | **Total** | **30.1** | **35.7** | **19,008** | **12.0** |

Life expectancy at 50 years old for males and females corresponds to the one we computed for our cohort by region of residence. GDP per capita in euros corresponds to the year 2003, the first of our period of observation (Source: INE). Unemployment rate corresponds to the first trimester of 2003 (Source: INE).

# Adjustment of probabilities of dying

Because the population and mortality data we employ are not linked, the quantities $P_{R,B}(x,s,t)$ and $D_{R,B}(x,s,t)$ are not in a one to one correspondence due to yearly migration flows. That is, death and out-migration or in-migration are concurrent (competing) events and, to compute an unbiased conditional probability of dying for a given one-year interval, we must introduce some adjustments. The probability of joint occurrence of both events is the product of $q'_{R,B}(x,s,t)$ and $mg_{R,B}(x,s,t)$, where $q'_{R,B}(x,s,t)$ is the real, corrected death probability, and $mg(x,s,t)$ is the net migration rate during the year[1]. If we assume that both events are uniformly distributed over the one year interval, the number of (adjusted) deaths $D_{R,B}(x,s,t)$ and population $P_{R,B}(x,s,t)$ can be related as defined in expression S1.

$$D_{R,B}(x,s,t) = P_{R,B}(x,s,t) \times q'_{R,B} \times \left(1 - \frac{mg_{R,B}}{2}\right) \qquad (S1)$$

The relationship between the populations at the beginning and end of the time interval can be expressed as equation S2.

$$P_{R,B}(x+1,s,t+1) = \left(1 - q'_{R,B}\right) \times \left(1 - mg_{R,B}\right) \times P_{R,B}(x,s,t) \qquad (S2)$$

From equations S1 and S2 we obtain the corrected probability of dying:

$$q'_{R,B}(x,s,t) = q_{R,B} + A_{R,B} - \sqrt{q_{R,B} \times \left(q_{R,B} + S_{R,B} - 1\right) + A_{R,B}^2} \qquad (S3)$$

where $q_{R,B}$ and $S_{R,B}$ are the crude death and survival probabilities directly computed from the *Padron*:

[1] This rate can be positive (if there is a net out-migration) or negative (if there is net immigration).

$$q_{R,B}(x,s,t) = \frac{D_{R,B}(x,s,t)}{P_{R,B}(x,s,t)} \qquad (S4)$$

$$S_{R,B}(x,s,t) = \frac{P_{R,B}(x+1,s,t+1)}{P_{R,B}(x,s,t)} \qquad (S5)$$

and $A_{R,B}$ is defined as:

$$A_{R,B}(x,s,t) = \frac{1}{2} \times \left(1 + S_{R,B}\right) \qquad (S6)$$

Using these expressions we can compute the corrected age and sex specific conditional probabilities of dying, $q'_{R,B}(x,s,t)$, from 2003 to 2019 and for all combinations of regions of birth and residence. This gives 135,762 computed death probabilities. We then estimate the cohort populations at each time t, $P'_{R,B}$, as follows:

$$P'_{R,B}(x+t-t_0,s,t) = P_{R,B}(x,s,t_0) \times \prod_{a=0}^{t-t_0-1} \left(1 - q'_{R,B}(x+a,s,t_0+a)\right) \qquad (S7)$$

When $t = t_0$ (year 2003), we set:

$$P'_{R,B}(x,s,t_0) \equiv P_{R,B}(x,s,t_0) \qquad (S8)$$

The number of deaths in the period under examination is:

$$D'_{R,B}(x,s,t) = P'_{R,B}(x+1,s,t+1) - P'_{R,B}(x,s,t) \qquad (S9)$$

We then pool these conditional corrected probabilities of dying $q'_{R,B}(x,s,t)$ in order to get the $q_{R,B}(x,s)$ and compute the rest of the life table functions by sex, region of birth and region of residence. This procedure is detailed in the main text.

# Alternative strategies to construct a pooled life table for the period under observation

The following is a description of alternative strategies to compute a single estimator of $e_{50}$ for each cell of the 11x11 place of birth and residence matrix.

*i. Standard actuarial method*

In each cell we compute mortality rates for age groups [50 +k,51+k) to [81+k, 82+k] and for the k[th] year $(0 \leq k \leq 17)$ after the onset of observation (year 2003). Each of these rates is computed using the ratios $D_{R,B}\,(x,s,t)/P^{*}_{R,B}$ (x,s,t) where $P^{*}_{R,B}$(x,s,t) is the average exposure, namely, the arithmetic average of $P_{R,B}(x,s,t)$ and $P_{R,B}(x+1,s,t$+1). We estimate 17 sets of mortality rates, one per year under observation, and convert them into conditional probabilities of dying using standard actuarial procedures (assuming uniformly distributed deaths, cohort homogeneity, etc…). These conditional probabilities are then used to build (left censored at age 50+k) life tables for each year k. Finally, we combine these life tables using weights that for each group [50+k,51+k) in the year 2003+k is equal to the fractional contribution represented by the population of the cohort that attained age 50+k in the year 2003+k. The result is a single sequence of (weighted) conditional probabilities that can then be chained together to construct a single life table representing the mortality experience after age 50 for the entire period for each cell of the place of birth x place of residence matrix.

*ii. Poisson model: option to identify cohort or period effects*

We model deaths counts for each age interval in each period, $D_{R,B}\,(x,s,t)$, as a Poisson random variable with offset $P'_{R,B}$(x,s,t). The includes dummies for age or, alternatively,

a function of age that reproduces the curvature of the mortality rates with age (a Gompertz, a Weibull, alternative). The model may/may not include dummies for the period to which the counts refer to or the cohort (defined at the outset of observation, in 2003) that contributes to the death counts and exposure (offset). Once the model parameters are estimated one can compute predicted values for the rates and, from them, life tables from age 50 for each year under observation. Finally, one can pool these using weights proportionally to the contribution to the exposure counts for year under observation.

*iii. Gompertz model*

Each cohort aged $50 \leq x \leq 68$ at the outset contributes to mortality rates $m_{x+k}$ , k=0, 1,…16. Cohorts aged $y \geq 69$ at the outset contribute to mortality rates $m_y$, $m_{y+1}$,…$m_{85+}$

There will be one observation for mortality rate $m_{50}$, two for mortality rate $m_{51}$, three for mortality rate $m_{52}$ and so on up until $m_{69}$ for which we will have 16 observations. By the same token, there will be two observations for mortality rate $m_{70}$, three for mortality rate $m_{71}$ etc…until 16 mortality rates for $m_{85+}$. We can then estimate the following Gompertz model :

$$\ln(m_x(t)) = \alpha + \beta x + \gamma t$$

where $t$ is the year to which the rate corresponds to (or a dummy for year or some other metric for year). We then predict $m_x(t)$ using the mean value of t for all x from 50 to 82, transform the predicted rates into probabilities, and build a single life table.

*iv. Logistic model*

Rather than working with observed mortality rates, one could focus on probabilities of surviving for all cohorts from ages $x$ to ages (x + min(16,85-x)) and $x \geq 50$. We can then estimate a logit model of the following form:

$logit\ S(x, \min(\ 82, x + 16)) = \alpha + \beta logitS(x, x + \min(82, x + 16))^{ST}\ + \delta Z, (\ 50 \leq x \leq 82)$

where $S(x, \min(\ 82, x + 16))$ is the observed probability of surviving from x to the maximum age we can observe for a cohort and $S(x, x + \min(82, x + 16))^{ST}$ is the same probability in a standard mortality pattern. Z is a vector of covariates that may include period or cohort identification. Once the parameters of the model are estimated, a unique life table can be computed. Note that methods (iii) and (iv) can generate estimates of period OR cohort effects that could be used explicitly if so desired. The approach we use in this paper ignores period/cohort changes and, as is done in methods (i) and (ii), we compute a pooled life table

# Quasi-Poisson model for mortality

To assess the effect of place of birth and residence on mortality (ignoring selection effects), we fitted a Poisson model with overdispersion (quasi-Poisson model) to the observed mortality rates. The model uses the log of the rates as dependent variable, population as offset, and includes controls for sex, age, the interaction of sex with age, and year of death. The model is defined by the following equation:

$$\log\big(\mathbb{E}(D)\big) = \log(P) + \beta_0 + \sum_{i=1}^{r} \beta_i \times B_i + \sum_{i=1}^{r} \beta_{r+i} \times R_i + \beta_{2r+1} \times age + \beta_{2r+2} \times S_f + \beta_{2r+3} \times age \times S_f + \beta_{2r+4} \times Y \qquad (S10)$$

where $\mathbb{E}(D)$ is the expected number of deaths in an interval, $P$ the exposed population, $B_i$ and $R_i$ are dummy variables for regions of birth and of residence, $S_f$ is a dummy variable set to 1 for females, and $Y$ the year of death. Thus, the ratios of mortality rates (MRR) are the exponentiated coefficients β from the model. The fit of the model is assessed by $R^2$ (computed as 1 minus the ratio of residual deviance over null deviance) and has a value of 0.86. MRRs for each region of birth and residence are show in Figure S2. The selection effects are captured by the birth and residence terms. The MRRs values are quite consistent with results discussed in Section 3 of the paper. In particular, Madrid (MD) is the region of residence with the lowest mortality while Andalusia has the highest. Similarly, Castilla y Leon, Aragon and Galicia experience the lowest mortality by place of birth while Canarias has the highest, followed by Andalusia and Madrid. Note that the cases of Canarias and Madrid are quite singular. On one hand, Madrid exhibits the highest life expectancy as a place of residence but one of the three lowest by place of birth. On the other hand, Canarias ranks in an intermediate position

in terms of mortality by place of residence but is the worst performer for mortality  by place of birth. This type or correspondences are likely due to health selection effects (healthy immigrant entering Madrid and healthy outmigrants exiting Canarias).

Figure S2: MRRs from the quasi-Poisson model defined by eq. S10 by region of birth and residence, with 95 % confidence intervals (Catalonia is the reference for both).

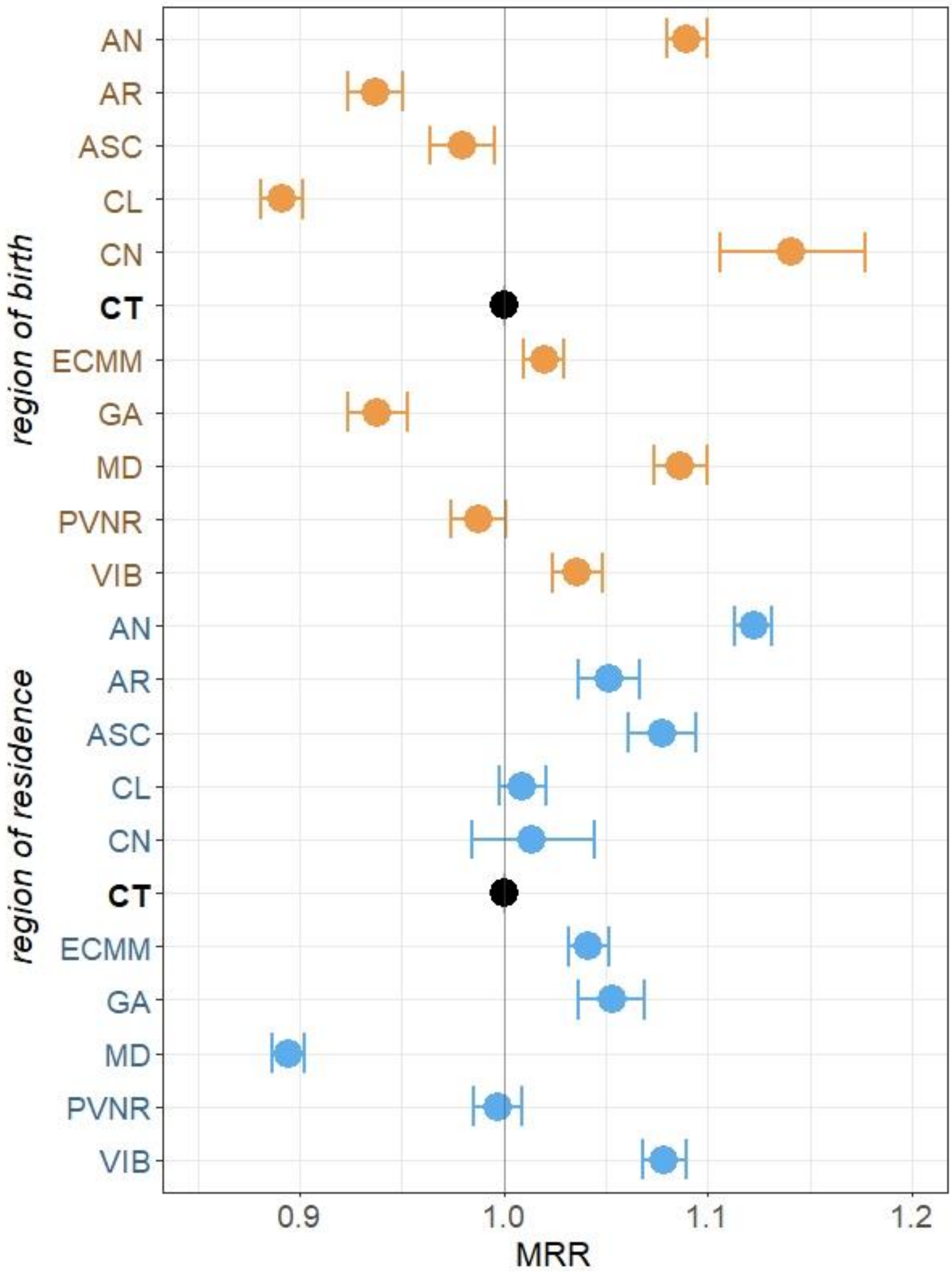

Supplement: Supplementary file 1 — Supplementary file1 (PDF 538 kb) [file 10680_2023_9679_MOESM1_ESM.pdf]
